# Game theoretic centrality: a novel approach to prioritize disease candidate genes by combining biological networks with the Shapley value

**DOI:** 10.1186/s12859-020-03693-1

**Published:** 2020-08-12

**Authors:** Min Woo Sun, Stefano Moretti, Kelley M. Paskov, Nate T. Stockham, Maya Varma, Brianna S. Chrisman, Peter Y. Washington, Jae-Yoon Jung, Dennis P. Wall

**Affiliations:** 1 Department of Biomedical Data Science, Stanford University, Stanford, USA; 2 Department of Pediatrics, Stanford University, Stanford, USA; 3 LAMSADE, CNRS, Université Paris-Dauphine, Université PSL, Paris, France; 4 Department of Neuroscience, Stanford University, Stanford, USA; 5 Department of Computer Science, Stanford University, Stanford, USA; 6 Department of Bioengineering, Stanford University, Stanford, USA; 7 Department of Psychiatry and Behavioral Sciences, Stanford University, Stanford, United States

**Keywords:** Coalitional game theory, Biological network, Shapley value, Game theoretic centrality, Autism spectrum disorder

## Abstract

**Background:**

Complex human health conditions with etiological heterogeneity like Autism Spectrum Disorder (ASD) often pose a challenge for traditional genome-wide association study approaches in defining a clear genotype to phenotype model. Coalitional game theory (CGT) is an exciting method that can consider the combinatorial effect of groups of variants working in concert to produce a phenotype. CGT has been applied to associate likely-gene-disrupting variants encoded from whole genome sequence data to ASD; however, this previous approach cannot take into account for prior biological knowledge. Here we extend CGT to incorporate a priori knowledge from biological networks through a game theoretic centrality measure based on Shapley value to rank genes by their relevance–the individual gene’s synergistic influence in a gene-to-gene interaction network. Game theoretic centrality extends the notion of Shapley value to the evaluation of a gene’s contribution to the overall connectivity of its corresponding node in a biological network.

**Results:**

We implemented and applied game theoretic centrality to rank genes on whole genomes from 756 multiplex autism families. Top ranking genes with the highest game theoretic centrality in both the weighted and unweighted approaches were enriched for pathways previously associated with autism, including pathways of the immune system. Four of the selected genes HLA-A, HLA-B, HLA-G, and HLA-DRB1–have also been implicated in ASD and further support the link between ASD and the human leukocyte antigen complex.

**Conclusions:**

Game theoretic centrality can prioritize influential, disease-associated genes within biological networks, and assist in the decoding of polygenic associations to complex disorders like autism.

## Background

The advent of next-generation sequencing technologies has rapidly decreased the cost of sequencing genomes and increased the throughput exponentially, making it possible to amass large amounts of data for conducting genome-wide association studies (GWAS) [1, 2]. Despite the abundance of high resolution genomic data, traditional GWAS approaches have faced mathematical and computational challenges in identifying candidate genes in diseases with complex genetic etiology.

Coalitional game theory (CGT) has been proposed as a novel and powerful way to identify candidate genes and assess their relevance to a given condition [3–5]. CGT studies the interaction of players–in our case genes–involved in a game by evaluating the coalitions that form and finding players that marginally contribute the most on average. More recently, CGT has been applied to fully sequenced genomes to assess the impact of groups of variants on phenotype and has previously been used to implicate likely gene disrupting (LGD) variants in Autism Spectrum Disorder (ASD) [6, 7]. However, these previous applications are unable to combine a priori biological knowledge like pathway information and autism genes of interest.

Incorporating such biological information between genes into analyses has improved the accuracy of predictors through pathway-based feature selection and aided genome-wide prediction of autism risk genes with limited genetic evidence using a human-brain gene network [8, 9]. Exploring the topological properties of biological networks has also been proposed as a way to study the combinatorial effects of components in a biological system. For instance, removing nodes from the protein-protein interaction network of integrin activation in human primary leukocytes and measuring the change of centrality values successfully predicted the functional and regulatory relevance of proteins in the network [10].

In this paper, we extend the CGT method implemented in Gupta et. al (2017) by combining it with the neighborhood-based game theoretic centrality measure introduced in Cesari et al. (2017), allowing for the incorporation of a priori network knowledge [6, 11]. We apply the method to 1965 children from 756 multiplex families and find a network of candidate genes harboring variants that likely interact to increase ASD risk.

## Results

### Game theoretic centrality genes

Table 1 lists the genes that were selected at the 0.05 threshold for the game theoretic centrality analysis. 13 of the 48 genes in the second analysis overlap with CASh analysis genes, suggesting that combining network information does affect the relevance of a gene. Not surprisingly, the first analysis, which mostly relies on the well annotated genes with corresponding protein product available in STRING, does not rank pseudogenes highly.

**Table 1:** Table of selected genes

Analysis	Genes
First Analysis	A2M, NT5C1B, PGM1, ERCC1, H6PD, CCR5, VNN1, OAS3, FAM187B, FOLH1, COL6A5, ASB15, GALNT9, CYP2C19, PPIG, RAD52,
	IFIH1, WWTR1, DNAH11, FSIP2, PIK3C2G, GJE1, WDR63, SLC25A43, APOOL, HLA-B, HLA-G, HLA-A, OPRM1, HLA-DRB1, TLR8, EGF,
	PNLIPRP3, GRIA1, GUCY2F, LPL, CYP2D6, COL4A6, IL12RB1, CYP2C18, GSTT2B, PSG3, GLRA4, PSG1, GPR119, GPR142, ACYP2, PPP1R3F
Second Analysis	OR2T4, CTB-23I7.1, AP002856.6, SSPO, OR6C1, BPIFB5P, RP11-573D15.1, SCRN3, RP11-404K5.2, RP11-104E19.1, AC008703.1, PEBP4,
	CSAG1, LRRIQ1, OR4Q2, ERCC6L2, OR7E5P, ZNF473, KRTAP13-2, AC007680.2, OR52B4, AP000289.6, C11orf40, TMEM254-AS1,
	AC023115.1, MUC19, NOS2P1, PDE4DIP, VCX3A, RP11-780M14.1, CLECL1, GAB4, CCDC7, ST3GAL6-AS1, ZNF586, OR5H8P, PKD1L2,
	OR4L1, MAGEE2, AC007317.1, ATP6AP1, ATP6V1B1, OR51I2, RP11-613D13.4, GSDMB, GUCY2F, GUCA1C, PRSS48
CASh	A2ML1, AC008703.1, AC093911.1, ALOX15P2, ATP13A5, BORA, BPIFB5P, C12orf60, C3orf35, CARD8, CCDC26, CCDC7, CDH15,
	COQ10A, CTC-525D6.1, DUSP16, ERCC6L2, FAM151A, FAM81B, FLG, GBGT1, HLA-K, LGALS8, MAGEC3, MYCT1, OR2T4, OR4Q2,
	OR6C1, OR8B3, RBAK-RBAKDN, RP11-104E19.1, RP11-160N1.10, RP11-404K5.2, RP11-56H2.2, RP11-618I10.2, RP11-738O11.13,
	SLC3A1, SSPO, TCP11, TRBV6-7, TRIM48, UBXN11, YME1L1, ZNF99, AF196972.4, AP002856.6, ATP6V1B1, C10ORF68, CDRT15P1,
	CTB-23I7.1, CTD-2130O13.1, CTD-2509G16.2, GEN1, KRT43P, MDP1, MPRIP, NT5C1B, OR4P4, OR5M10, OR5M11, OR8I2, PRIM2,
	RP11-15E18.4, RP11-283G6.4, RP11-705C15.2, SSXP3, VWA7

Table of genes that were selected using the three different analyses described in the section, Game theory analyses

Incorporating the protein-protein interaction network led to genes that are biologically relevant to ASD and have not been previously identified through CASh analysis in Gupta et al. (2017). Mutation in X-linked ATP6AP1 has been shown to lead to immunodeficiency with cognitive impairment [12]. GUCA1C and GUCY2F are both in the pathway of signaling by GPCR, which has been implicated in neurodevelopmental disorders including ASD and Fragile X syndrome [13]. PDE4DIP has been identified as a putative target for brain-enriched miRNA, where PDE4DIP is a homolog of CDK5RAP2, a gene that has been linked to microcephaly [14].

**Fig. 1:**
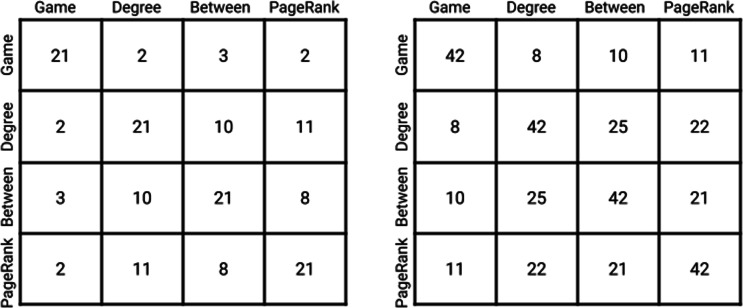
Common top-ranking genes among the centrality measures. Each element of the matrix represents the number of genes shared at the top 10% (left matrix) and 20% (right matrix) ranking between two centrality measures in comparison. The complete list of genes ranked by the various centrality measures can be found in “Additional File 1” under “*Supplementary information*”

We also ran commonly used centrality measures (degree centrality, betweenness centrality, PageRank algorithm) over the protein-protein interaction network. In order to make a comparable comparison between other centrality measures that only uses the connected graph and game theoretic centrality, we removed all the isolated genes ranked by game theoretic centrality. The ranking among degree centrality, betweenness centrality, and PageRank algorithm share close to 50% of the genes in pairwise comparisons, but the number of shared genes with game theoretic centrality is lower at around 10% to 20% as shown in Fig. 1. Among these shared genes, game theoretic centrality selected genes that are not necessarily of the highest rank in the other three measures. This suggests that the game theoretic centrality method is a novel centrality concept that incorporates trade-off between connectivity and weights of nodes, highly ranking genes that otherwise would not surface at the top. Furthermore, among the genes uniquely identified by game theoretic centrality at the highest 10% ranking, ATP6AP1, GUCY2F, and GUCA1C emerge at the top. These genes are shown to be previously implicated in ASD when game theoretic centrality is compared to CASh analysis. The full list of ranked genes can be found in “Additional File 1” under “*Supplementary information*”.

### Biological validation

In order to look for possible associations with ASD, we cross-referenced the top ranking genes from the first and second analyses with candidate ASD genes highlighted through previous publications. In particular we compared the top ranking game theoretic centrality genes with three different sources of candidate ASD genes–a curated list of known genes associated with ASD from Simon’s Foundation Autism Research Initiative (SFARI), a set of genes shown to be differentially expressed in blood and brain tissues of individuals diagnosed with ASD known as the Root 66 gene list, and a list of 69 genes harboring rare variants implicated for increased ASD risk [15–17]. GRIA1 is the only gene shared in both the first analysis and the 69 genes published in Ruzzo et al. (2019). Beyond looking for overlaps between the gene sets, we searched for protein-protein interactions between the game theoretic centrality genes and the known high confidence genes using STRING.

**Fig. 2:**
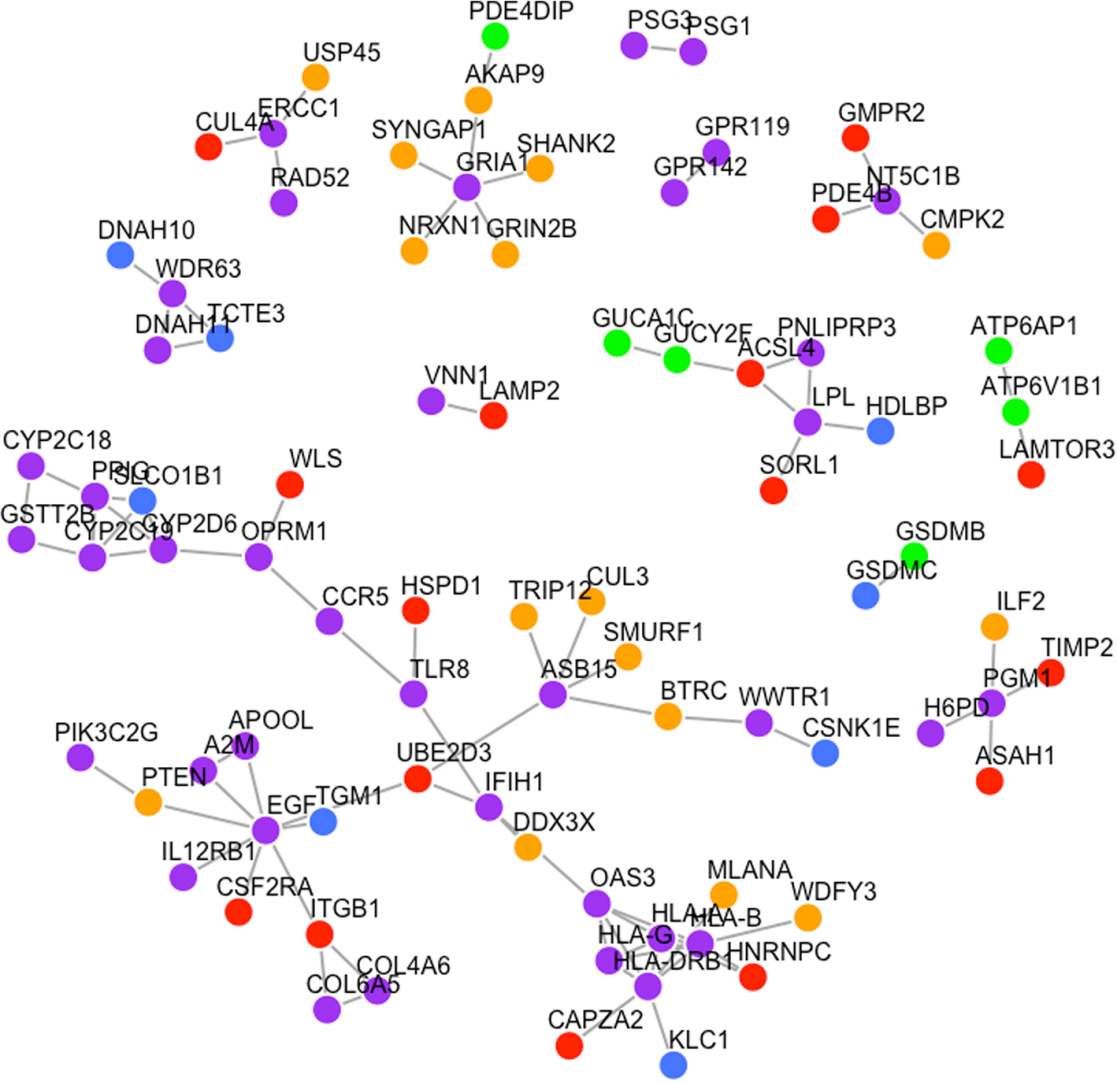
Graph of protein-protein interactions between game theoretic centrality genes and high confidence ASD genes. Node color: first analysis (purple), second analysis (green), SFARI (blue), 69 genes from Ruzzo et al. 2019 (yellow), Root 66 (red)

CASh analysis identified 9 genes with protein-protein interaction with high confidence candidate genes in Gupta et al. (2017). As shown in Fig. 2, the game theoretic centrality method identified 6 genes–distinct from CASh analysis genes–that have protein-protein interaction with high confidence candidate genes. While game theoretic centrality identified less genes with protein-protein interaction with candidate genes, 3 of the identified genes have been implicated in ASD or other neurodevelopmental disorders as shown in “Game theoretic centrality genes”. The first analysis identified 39 genes that are in protein-protein interaction with high confidence candidate genes.

We also checked for significant pathways in which the top ranking game theoretic centrality genes were enriched for using Reactome Pathway Browser (reactome.org), a database of known pathways and biological processes [18]. Reactome identified 27 significant pathways for the genes underscored through the first and second analyses of which the following pathways have been implicated in ASD in the past: Immune system (FDR =2.15×10^−15^), endosomal pathway (FDR =2.15×10^−15^), cytokine signaling in the immune system (FDR =2.15×10^−15^), olfactory signaling pathway (FDR =4.72×10^−2^), and insulin receptor recycling (FDR =7.06×10^−2^) [19–24].

Four of the genes ranked in the first analysis, HLA-A, HLA-B, HLA-G, and HLA-DRB1 belong in the human leukoctye antigen (HLA) complex and have been previously implicated in ASD [25]. HLA class I molecules have been shown to play a role in neural development and regulate activity-dependent refinement and plasticity [26, 27]. HLA-DRB1 has been linked with increased ASD risk possibly through gastro-intestinal and gut-brain axis dysregulation [28, 29].

Apart from the genes in the HLA complex, two of the ranked genes individually have been associated with ASD or other neurodegenerative diseases. For OPRM1, a monogenic mouse model experiment has shown that disrupting the mu-opiod receptor signaling can induce autistic behaviors in mice [30]. A2M is known to mediate the clearance of amyloid-beta, a protein product commonly elevated in individuals with Alzheimer’s disease [31]. Examining the postmortem brains of individuals diagnosed with ASD has shown abnormal accumulation of the amyloid-beta protein compared in the postmortem brains of neurotypical individuals [32].

## Discussion

We demonstrated that game theoretic centrality can uncover genes that may play an integral role in the complex regulatory activity of a network of genes in the context of ASD. Game theoretic centrality preferentially ranks genes that are connected to a large number of genes that themselves do not have many neighbors. Figure 2 shows that 39 genes from the first analysis and 6 genes from the second analysis have direct protein-protein interactions with high confidence ASD genes. This suggests that harboring variants in the high ranking game theoretic centrality genes may interact with the high confidence ASD genes through a regulatory framework. Note, however, that the approach may rank highly connected genes at the top that are in turn more likely to interact with the high confidence ASD genes by chance. Performing pathway analysis also showed that the top ranking genes are enriched in pathways of biological functions that have been previously linked with ASD, further corroborating the potential effect of these genes.

This study is limited to well-annotated protein-coding genes where gene to gene interaction networks like co-expression and protein-protein interaction data are available to build graphs. With mounting evidence for the importance of non-coding region in the genetic etiology of disorders like ASD, it is necessary to incorporate ways to include non-coding sequences in the analysis, allowing the exploration of interactions between the coding and non-coding space. In future works, game theoretic centrality can also be applied to computable networks representing various biological systems apart from protein level interactions and expanded to other curated databases. More functional studies of the top ranked genes are needed to further evaluate the role of these genes in ASD.

## Conclusion

In this study, we extended the analysis performed in Gupta et. al (2017) by applying a game theoretic centrality measure based on Shapley value to rank genes by their relevance to a condition. While the previous work focuses on the frequency of co-alteration of LGD mutations, we created a framework to integrate known biological networks independent of the data set to the analysis. Both studies aim to take into account the combinatorial interactions between genes beyond the effect of each individual gene to a given phenotype that classical GWA studies generally target. We showed that game theoretic centrality and neighborhood-based relevance index can select candidate genes that have been associated with ASD suggesting that highly ranked genes that have not been previously linked with ASD may also play a critical role.

Game theoretic centrality, characterized by its capacity to capture combinatorial interaction between genes and integrate a priori knowledge, is a compelling tool for prioritizing candidate genes. Unconventional and novel approaches like game theoretic centrality can ultimately contribute to the development of translational research and facilitate the discovery of clear biomarkers for complex human health conditions like ASD.

## Methods

### Coalitional game theory

Coalitional game theory aims to model the interaction of players in a game and various ways to allocate the payoff among the players, or to measure their importance. More formally, a coalitional game is defined as a pair (*N*,*v*), where *N* is a finite set (of players) and *v*:2^*N*^→*R* represents a *characteristic function* that maps a positive real-valued number *v*(*T*)∈*R* to each *coalition* *T*⊆*N* (we assume *v*(*∅*)=0 and *v*(*N*)=1). The Shapley value is a popular *solution* for such coalitional games, commonly employed across various disciplines like economics and political science [33]. The Shapley value *ϕ*_*i*_(*v*) of a player *i*∈*N* in *v* is defined as its average marginal contribution across all possible permutations of players, and is computed as follows:

1 $$ \begin{aligned}  \phi_{i}(v) = \sum\limits_{T \subseteq N, i \in T}\frac{(|N|-|T|)!(|T|-1)!}{|N|!}\big(v(T)-v(T\setminus\{i\})\big) \end{aligned}  $$

where *v*(*T*)−*v*(*T*∖{*i*}) is the marginal contribution of player *i* to coalition *T*, with *T*⊆*N* and *i*∈*T*, and $\frac {(|N|-|T|)!(|T|-1)!}{|N|!} $ is the probability that player *i* joins coalition *T*∖{*i*} according to a mechanism that randomly selects (with a uniform probability distribution) a permutation of the elements of *N*.

### Microarray game

**Fig. 3:**
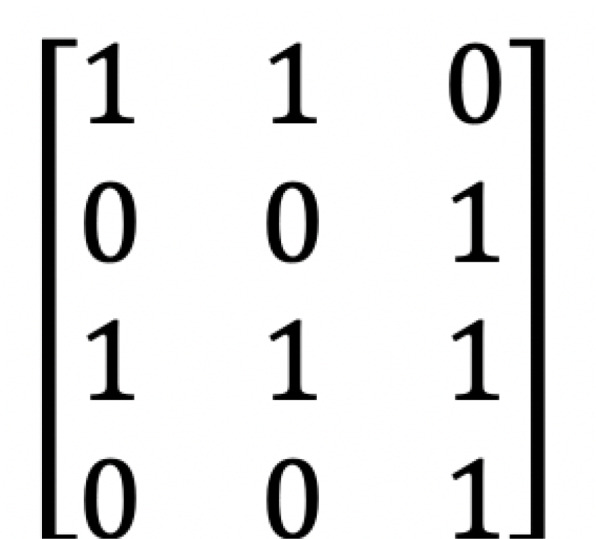
Example 4x3 binary matrix. 4×3 binary matrix representing 4 genes and 3 samples

Let *B*∈{0,1}^*n*×*m*^ be a binary matrix with *N*={*g*_1_,*g*_2_,...,*g*_*n*_} genes and *S*={*s*_1_,*s*_2_,...,*s*_*m*_} samples (with the convention that *B*_*ij*_=1 represents the presence of a feature such as abnormal expression or the presence of a loss of function mutation for a given gene *g*_*i*_∈*N* and sample *s*_*j*_∈*S*, whereas *B*_*ij*_=0 represents the absence of such feature) [6, 34]. Given a coalition *T*⊆*N*, consider the unanimity game (*N*,*u*_*T*_) defined such that

$$ u_{T}(W)=\left\{\begin{array}{cc} 1, & \text{if}\ T \subseteq W \\ 0, & \text{otherwise} \end{array}\right. $$

for any *W*⊆*N*. Introduced in the paper Bonassi et. al (2007), a microarray game is a coalitional game (*N*,*v*^∗^) based on the binary matrix *B* and a characteristic function expressed in terms of a linear combination of unanimity games,

2 $$ v^{*}(T)=\frac{1}{|S|} \sum\limits_{j\in S} u_{M_{j}}(T)=\frac {|{j\in S : M_{j} \subseteq T}|}{|S|}  $$

where *M*_*j*_⊆*N* is the set of genes with present features (*B*_*ij*_=1) for *j*∈*S* and each column is a unanimity game. *v*^∗^(·) measures the frequency of the genes in coalition *T* showing the same properties across experiments or samples. For example in Fig. 3, the value of coalition *g*_1_,*g*_3_ is given by $v^{*}({g_{1},g_{3}}) = \frac {2}{3}$.

Computing the Shapley value can become computationally intractable as the number of players *N* increases and must iterate through all 2^*N*^ coalitions. The paper Bonassi et al. (2007) introduces an approach to calculate the Shapley value *ϕ*(*v*^∗^) for each player *i*∈*N* in polynomial time, reducing relations (1) and (2) to

3 $$ \phi_{i}(v^{*}) = \sum\limits_{j \in S} \frac{R_{ij}}{|S|}  $$

where,

$$ R_{ij}=\left\{\begin{array}{cc} 0, & \text{if}\ B_{ij} = 0 \\ \frac{1}{|M_{j}|}, & \text{otherwise} \end{array}\right. $$

for any *g*_*i*_∈*N* and *s*_*j*_∈*S*. For the microarray game defined on the boolean matrix of Fig. 3, genes *g*_1_,*g*_2_,*g*_3_,*g*_4_ get the following Shapley values using relation (3), $\left (\frac {3}{9}, \frac {1}{9}, \frac {4}{9}, \frac {1}{9}\right)$. *g*_2_ and *g*_4_ have the same pattern and consequently get the same value.

### Game theoretic neighborhood-based relevance index

**Fig. 4:**
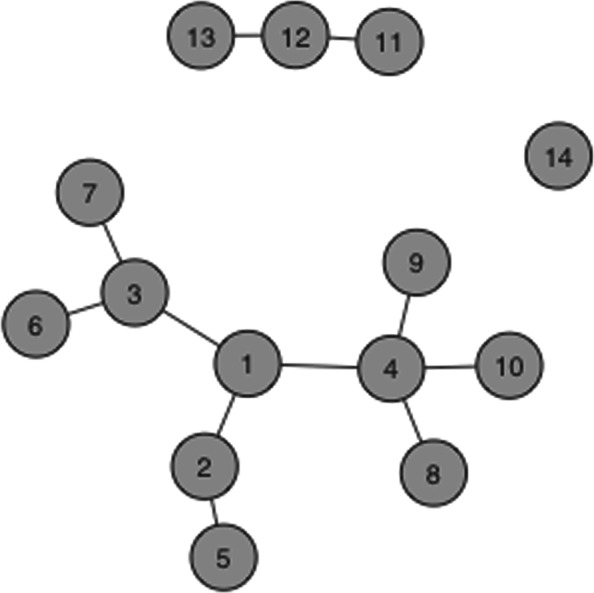
Example network. A graph of 14 vertices (genes) *N*={*g*_1_,*g*_2_,...,*g*_14_} and 11 edges (respective biological interactions)

The graph 〈*N*,*E*〉 is a network where *N* represents a set of genes and *E* a set of edges connecting the genes. An edge {*g*_*k*_,*g*_*l*_}∈*E* between two nodes describes an interaction between the two genes *g*_*k*_,*g*_*l*_∈*N* (to avoid cumbersome notations, later we will denote an edge {*g*_*k*_,*g*_*l*_} as *g*_*k*_*g*_*l*_). The parameter vector *k*∈*R*^*N*^ assigns a weight based on a priori knowledge for each of the genes *i*∈*N*. If each element of the parameter vector *k* is set to 1, then each of the nodes are weighted equally and no a priori knowledge is incorporated into the graph. The coalitional game corresponding to the graph 〈*N*,*E*〉 is defined by $\left (N, v^{k}_{E}\right)$ where the characteristic function is defined as,

4 $$ v^{k}_{E}(T) = \sum\limits_{j \in T\cup N_{T}(E)}k_{j}  $$

where *N*_*T*_(*E*) is set of nodes that are adjacent to the nodes in *T*⊆*N*. For any coalition *T*⊆*N*, $v^{k}_{E}(T)$ takes the sum of all the a priori weights *k*_*j*_ for *j*∈*T*∪*N*_*T*_(*E*). For example, in Fig. 4, suppose each node has a weight of *k*_*i*_=1, for each *i*∈*N*, then the value of the coalition *T*={12,13,14} is $v^{k}_{E}(\{T\})=4$, as *T*∪*N*_*T*_(*E*)={11,12,13,14}.

Similar to computing the Shapley value *ϕ*(*v*^∗^) for microarray games, the Shapley value $\phi \left (v^{k}_{E}\right)$ becomes computationally intractable with growing number of players introduced in the game using the classical Shapley value formula in relation (1). The paper Cesari et al. (2017) axiomatically characterizes the Shapley value on the class of games defined by relation (4) and proves for each player *i*∈*N* the Shapley value $\phi \left (v^{k}_{E}\right)$ can be computed in polynomial time using the following equation,

5 $$ \phi_{i}\left(v^{k}_{E}\right) = \sum\limits_{j \in (N_{i}(E) \cup \{i\})}\frac{k_{j}}{deg_{j}(E)+1}  $$

where *deg*_*j*_(*E*) is the *degree* of node *j*, i.e the cardinality of the set of edges *E* connected to node *j*, and *N*_*i*_(*E*) is the set of nodes connected to *i* [11]. For the network depicted in Fig. 4, the vector of genes’ centrality values assuming *k*_*i*_=1 for all *i*∈*N* and using relation (5) is $\left (\frac {62}{60},\frac {65}{60},\frac {90}{60},\frac {117}{60},\frac {50}{60},\frac {45}{60},\frac {45}{60},\frac {42}{60},\frac {42}{50},\frac {42}{60},\frac {50}{60},\frac {80}{60},\frac {50}{60},\frac {60}{60}\right)$. Note that a gene will achieve a higher score if the node is connected to many nodes that themselves have a small number of neighboring nodes. For instance, while *g*_1_ is more central in the network, *g*_4_ which is connected to 3 nodes that themselves do not have neighbors has a greater score. Removing the edges incident to *g*_1_ from the network would cause the component containing 1 to split into four components with one isolated component, {{*g*_1_},{*g*_3_,*g*_6_,*g*_7_},{*g*_2_,*g*_5_},{*g*_4_,*g*_8_,*g*_9_,*g*_10_}}. While removing the edges incident to *g*_4_ would split the same component into five components {{*g*_4_},{*g*_8_},{*g*_9_},{*g*_10_},{*g*_1_,*g*_2_,*g*_3_,*g*_5_,*g*_6_,*g*_7_}}, and this would leave four components isolated, consequently affecting the regulatory activity of more genes. More examples can be found in the paper Moretti et al. (2018) comparing game theoretic neighborhood-based relevance index to other commonly employed centrality measures such as degree and betweenness centrality.

### Game theoretic centrality: a combined approach

**Fig. 5:**
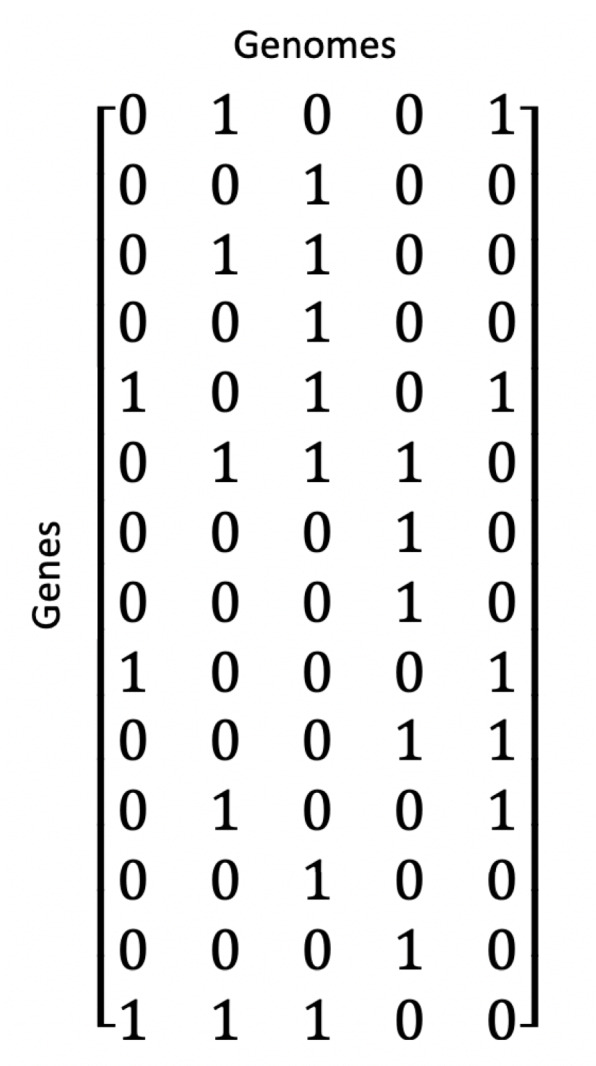
Example 14x5 binary matrix. 14×5 binary matrix representing 14 genes and 5 samples

**Fig. 6:**
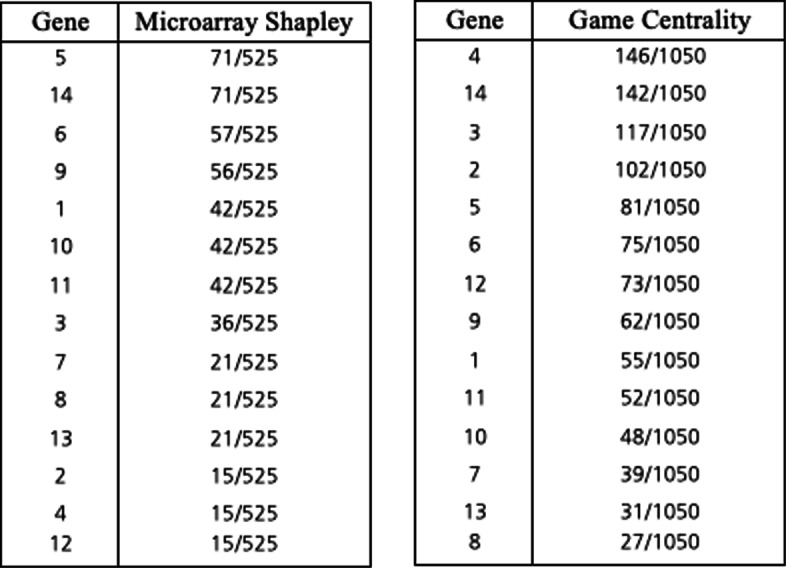
Example gene ranking. Table A. (left) shows the gene ranking based on microarray game. Table B. (right) shows the gene ranking based on game theoretic centrality with microarray Shapley values as weights. The genes are sorted by highest (top) to lowest (bottom) score

Microarray games have been used to rank genes based on the frequency of specific coalitions across samples given an observed data of microarray experiments, taking into account the interaction of genes within coalitions with equal weight. By incorporating microarray game results into game theoretic neighborhood-based relevance index as defined by relation (5) through the parameter vector *k*∈*R*^*N*^, we can take into account for known biological interactions that have been studied extensively and give more weight to certain coalitional interactions. As a consequence, outliers that are unlikely due to true gene interactions, but rather random associations that can be attributed to chance, can be removed. Consider a microarray game (*N*,*v*^∗^) corresponding to the binary matrix *B*^14×5^ presented in Fig. 5. Let each row of *B* represent a gene in *N*={1,2,...,14} and each column represent an individual. The value *B*_12_=1 indicates that second individual has at least one loss of function mutation in gene 1. Computing the Shapley value from the binary matrix using relation (3) yields the ranking shown in Table A of Fig. 6. It is possible for a gene with low Shapley value on microarray games to play a critical role in the regulatory activity of a group of genes with high Shapley values. In addition to the microarray game from the binary matrix, now consider the game $\left (N, v^{k}_{E}\right)$ corresponding to the graph 〈*N*,*E*〉 shown in Fig. 4. Instead of setting the parameter vector *k* to a vector of ones, we assign the microarray Shapley values (*ϕ*_1_(*v*^∗^),*ϕ*_1_(*v*^∗^),...,*ϕ*_14_(*v*^∗^)) to *k*. Computing the game theoretic centrality using relation (5) results in the ranking shown in Table B of Fig. 6. Notice that gene 4 and gene 12 were initially ranked low based on the results from the microarray game; however, once the Shapley values are incorporated into game theoretic centrality as node weights, gene 4 and gene 12 rise towards the top. While gene 4 and gene 12 have low microarray Shapley values, they are connected to multiple genes that themselves do not have neighboring genes as shown in Fig. 4. In contrast, gene 14 retains the same rank despite being disconnected from the graph. Gene 14 does not have a direct interaction defined by the network, but the empirical evidence for synergistic effect captured by the microarray game maintains a high score and suggests a potential unknown interaction important in regulating this group of genes. This example demonstrates the motivation for combining the two games and their respective Shapley values in a single measure that better represents the effective overall connectivity of a gene in a network. The combined approach provides a novel mechanism for balancing the relevance of a gene to a phenotype from empirical data as well as known biological models.

### Data preprocessing

We applied game theoretic centrality to 30x coverage whole genome sequencing data from the Hartwell Foundation’s Autism Research and Technology Initiative (iHART). The iHART initiative is a collaborative effort to amass fully sequenced genomes of multiplex families with two or more children diagnosed with autism. Specifically, we analyzed 1,965 genomes–1,616 children diagnosed with ASD and 349 unaffected children–and removed all non-Mendelian mutations to exclude de novo mutations and possible sequencing artifacts, which may lead to spurious signals. We further filtered for genes with highest predicted impact–likely gene disruption (LGD)–and only included loss-of-function mutations with high haplotype-aware consequences (CSQ impact). We encoded these genomes into two binary matrices *B*^*case*^ and *B*^*control*^, where 1 represents the presence of at least one homozygous alternate LGD loss-of-function mutation or a compound heterozygous variant for a given gene, and 0 for reference. These preprocessing steps reduced the total number of genes from 13,853 to 965 genes, leading to final binary matrices with the dimension of 965 genes by 1,965 genomes. We then generated a protein-protein interaction network with the genes included in the binary matrices using STRING database V11 (string-db.org) [35]. STRING is a comprehensive database of known and predicted physical and functional protein-protein interactions obtained through multiple data sources including experimental evidence and text-mining. We filtered for interactions with confidence score ≥0.6, where a confidence score of 0.4 is considered medium confidence for a true interaction and 0.7 high confidence, producing a graph 〈*N*,*E*〉 with 965 vertices (genes) and 273 edges (protein-protein interactions). A slightly lower threshold of 0.6 was chosen to populate the graph with sufficient number of edges. The change in confidence score does not affect the rankings of the gene at the top five percent level for the game theoretic centrality method.

### Game theory analyses

For the first analysis, we apply game theoretic neighborhood-based relevance index without any a priori knowledge, i.e. *k*={1,1,...,1}, to the protein-protein interaction network of 965 genes using relation (5). We select the top five percent of genes with highest game theoretic centrality score.

**Fig. 7:**
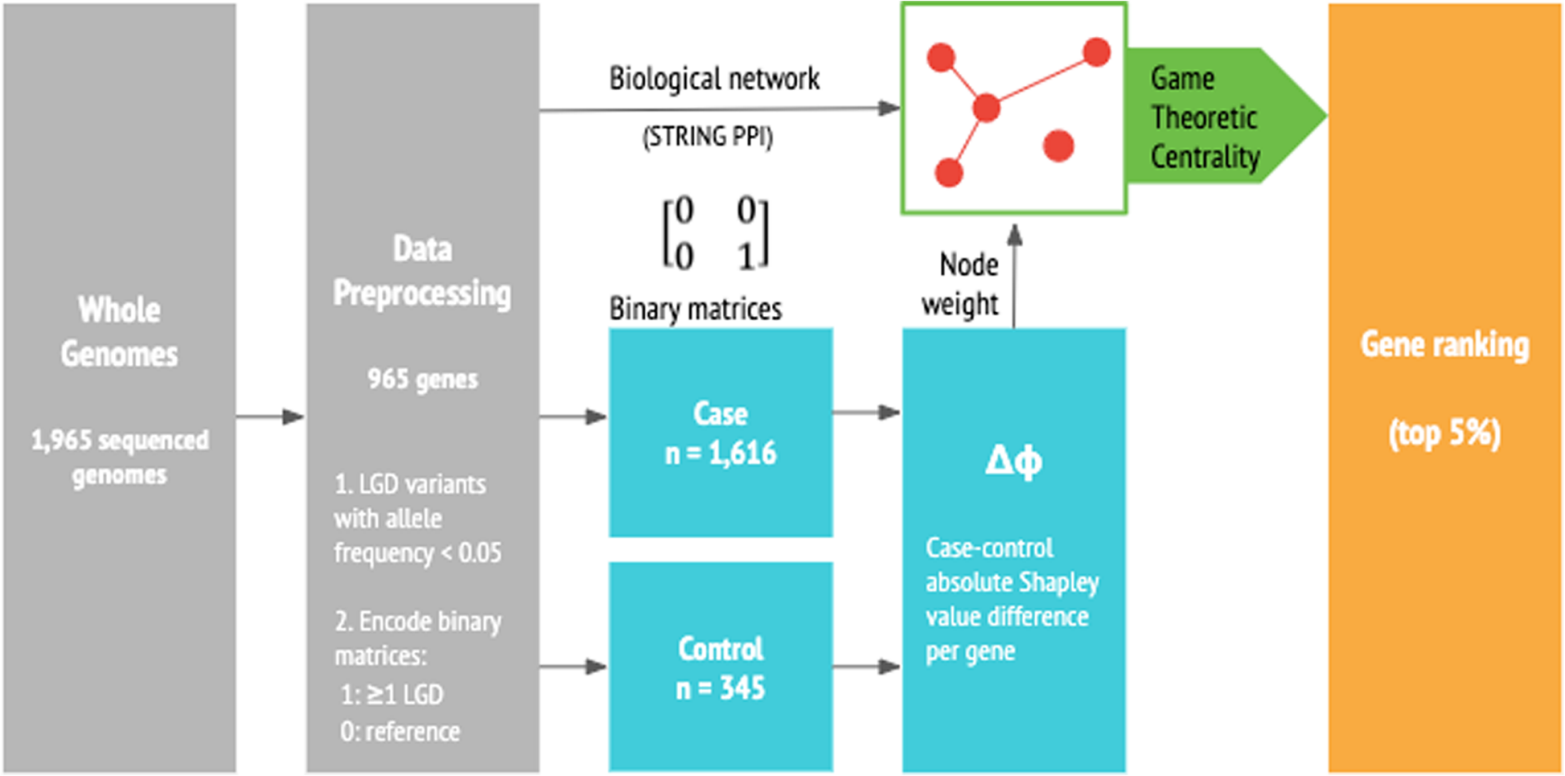
Game theoretic centrality flow diagram. Flow diagram, beginning from the whole genome sequence data to ranking genes using game theoretic centrality

For the second analysis, we apply game theoretic centrality with a priori weights as described in the “Game theoretic centrality: a combined approach” section using relation (5) and parameter vectors derived from the case and control binary matrices using relation (3). This produces two sets of ranking, each for case and control. For each gene *i*∈*N*, we consider the absolute difference of the game theoretic centrality value between the case and control ranking,

6 $$ \delta_{i} = abs\left(\phi_{i}\left(v^{case}_{E}\right) - \phi_{i}\left(v^{control}_{E}\right)\right)  $$

where the parameter *case* corresponds to the Shapley vector computed according to relation (3) on the microarray game defined over the case binary matrix, and *control* is the Shapley vector computed according to relation (3) on the microarray game over the control binary matrix. Figure 7 visualizes the game theoretic centrality approach applied to the whole genomes. The second analysis is similar to Comparative Analysis of Shapley Value (CASh) analysis introduced in Moretti et. al (2008), in that they both rank the genes based on the absolute difference of the scores between the case and control group. More specifically, CASh analysis computes the microarray game Shapley value between case and control group and selects the genes through a bootstrapping based multiple hypothesis testing procedure, thereby combining Shapley value with statistical significance. The paper Gupta et. al (2017) describes how CASh analysis was applied to the binary matrices of LGD variants described in the “Data preprocessing” section to select ASD candidate genes.

## Supplementary information

**Additional file 1** Full list of genes ranked by game theoretic centrality, degree centrality, betweenness centrality, and PageRank algorithm.

## Data Availability

The datasets generated and/or analyzed during the current study are available in the Hartwell Autism Research and Technology Initiative (iHART) repository upon approval by the Data Access Committee. http://www.ihart.org/access

## Abbreviations

GWAS: Genome-wide association study
ASD: Autism spectrum disorder
CGT: Coalitional game theory
LGD: Likely gene disrupting
CASh: Comparative analysis of Shapley value
HLA: Human leukocyte antigen
