# Supplementary material for: Game theoretic centrality: a novel approach to prioritize disease candidate genes by combining biological networks with the Shapley value
Source: BMC Bioinformatics. 2020 Aug 12;21:356. doi: 10.1186/s12859-020-03693-1 (PMC7430867; doi:10.1186/s12859-020-03693-1)
Supplement: Supplementary file 1 — Additional file 1 Full list of genes ranked by game theoretic centrality, degree centrality, betweenness centrality, and PageRank algorithm. [file 12859_2020_3693_MOESM1_ESM.pdf]

Rank	Game	Degree	Between	PageRank
1	ATP6AP1	HLA-B	FCGR2A	PGM1
2	ATP6V1B1	HLA-G	GP6	ERCC1
3	GUCY2F	HLA-A	TLR8	NT5C1B
4	GUCA1C	OAS3	COL4A6	A2M
5	CCHCR1	HLA-DRB1	PRPS1L1	VNN1
6	PSORS1C2	HLA-DQB1	CCR5	H6PD
7	ZNF880	HLA-DRB5	HLA-DRB1	FAM187B
8	C1orf194	HLA-DPB1	BTNL2	CCR5
9	CYP2D6	HLA-DQA1	IFNA10	PPIG
10	PGM1	TRIM48	PGM1	EGF
11	FUT2	PGM1	IL12RB1	OAS3
12	FUT6	A2M	LILRA1	OPRM1
13	ANKMY1	CCR5	EGF	RAD52
14	ANO7	H6PD	OPRM1	GALNT9
15	H6PD	OPRM1	CYP2D6	HLA-B
16	GBGT1	TLR8	H6PD	HLA-G
17	CC2D2A	EGF	LILRB4	HLA-A
18	CEP89	ERCC1	IFIH1	WWTR1
19	PYGL	IGF2	HLA-B	FSIP2
20	FMO2	CXCR3	HLA-G	PIK3C2G
21	LPL	APOOL	HLA-A	GJE1
22	GALNT9	IFIH1	OAS3	SLC25A43
23	ARPC5L	NT5C1B	PPIG	CYP2D6
24	ACTR3C	LILRA1	PYGL	DNAH11
25	EGF	RAD52	GLUD2	WDR63
26	SRA1	CYP2D6	TKTL2	ASB15
27	LILRA1	KRT83	A2M	APOOL
28	CPNE1	COL4A6	PHKA2	HLA-DRB1
29	CFL1	VNN1	ALDH1B1	GRIA1
30	LCP1	MMRN1	COL6A5	PNLIPRP3
31	VNN1	KRT26	CYP2C19	LPL
32	CCDC57	ASB15	APOOL	IGF2
33	GLT6D1	KRT24	CYP2C18	COL6A5
34	PHKA2	P2RY4	ACYP2	TLR8
35	IGF2	KRT31	PPP1R3F	PSG3
36	GEN1	TAS2R46	GUCY2F	GPR142
37	CYP2C19	FCGR2A	PDE6B	PSG1
38	CELA1	LILRB4	ACSL4	GPR119
39	PRIM2	NOD2	NT5C1B	GUCY2F
40	ZNF208	COL6A5	ERCC1	ACSL4
41	FSIP2	TKTL2	NOD2	CYP2C19
42	IFIH1	PPIG	CLEC7A	EFCAB13

43	MUC20	KRT37	PNLIPRP3	COL4A6
44	MUC7	XRCC4	LPL	CXCR3
45	ACYP2	PRPS1L1	RAD52	PDE6B
46	MTMR6	CYP2C19	VNN1	IFIH1
47	RAD52	PNLIPRP3	GEN1	HLA-DQB1
48	NOD2	GRIA1	GRIA1	HLA-DRB5
49	CCR5	UBE2W	GRIN3B	HLA-DPB1
50	CLEC7A	FLG2	FAM187B	HLA-DQA1
51	OAS3	FOLR3	XRCC4	TRIM48
52	WWTR1	SPSB2	TLR5	GEN1
53	PPIG	LDHB	TAAR2	GMPR
54	TLR8	GALNT9	GLRA4	TKTL2
55	BTNL2	ACSL4	GMPR	GLRA4
56	PSG3	GEN1	ASB15	XRCC4
57	ALDH1B1	TLR5	DNAH11	MMRN1
58	COL4A6	PDE6B	WDR63	ACYP2
59	MMP2	DNAH11	EFCAB13	TAAR2
60	OPRM1	CLEC7A	CHD1L	IL12RB1
61	SLFN12L	GRK4	PSG3	UBE2W
62	KISS1	FAM187B	GALNT9	SPSB2
63	GPRC6A	IL12RB1	PSG1	ZNF645
64	GRP	WDR63	GPR119	PYGL
65	AKR1E2	GP6	GPR142	PPP1R3F
66	DHDH	PYGL	IGF2	GRIN3B
67	ZNF681	MMP2	WWTR1	ATP6AP1
68	OR52N4	GUCY2F	FSIP2	FUT2
69	AKAP3	GMPR	PIK3C2G	CC2D2A
70	MMRN1	LPL	GJE1	LCE6A
71	NPPA	ZNF645	SLC25A43	IDO2
72	LILRB4	CHIT1	CXCR3	ATP8B4
73	APOOL	TRAPPC2	APLF	ARPC5L
74	LOXHD1	MUC20	HLA-DQB1	CFL1
75	TMPRSS3	EME2	TRAPPC2	PLXNB3
76	OR7G3	WWTR1	HLA-DRB5	L1TD1
77	DYX1C1	PHKA2	ATP6AP1	AKR1E2
78	PRR21	AMPD1	FUT2	SVOPL
79	PRR25	POLN	MUC20	SRPK3
80	PIK3C2G	CHD1L	GUCA1C	MAGEC1
81	GLRA4	MUC7	HLA-DPB1	PRR21
82	ABCB5	KISS1	EME2	MICA
83	SLC22A14	GPRC6A	CC2D2A	CCDC57
84	TAAR2	COL16A1	AMPD1	CCHCR1
85	PLXNB3	TAAR2	LCE6A	ABCB5

86	SEMA4C	BTNL2	POLN	POMT1
87	PSG9	PSCA	KRT83	ZNF681
88	EFCAB13	PSG3	IDO2	LOXHD1
89	PTH2R	CYP2C18	HLA-DQA1	ZNF157
90	COL6A5	GLRA4	MMRN1	ZNF880
91	LCE6A	PSG1	MUC7	YME1L1
92	LCE1D	GPR119	KISS1	OR13A1
93	DNAH14	GPR142	KRT26	ANKMY1
94	IL12RB1	FSIP2	GPRC6A	MLKL
95	C4orf17	ALDH1B1	COL16A1	KRT83
96	GMPR	PIK3C2G	KRT24	KRT26
97	GP6	ACYP2	SRA1	KRT24
98	TKTL2	GJE1	P2RY4	ATP6V1B1
99	GRIN3B	EFCAB13	KRT31	FUT6
100	ERCC1	DNAH14	TAS2R46	CEP89
101	ALDH1A2	TRAPPC2L	UBE2W	LCE1D
102	CHD1L	APLF	FLG2	INMT
103	TCTEX1D1	CEACAM7	ATP8B4	FRMPD3
104	UPP2	GRP	TRIM48	ACTR3C
105	HLA-DRB1	GRIN3B	FOLR3	LCP1
106	MLKL	GLUD2	SPSB2	SEMA4C
107	CHMP4C	SLC25A43	PSCA	DPPA4
108	OR13A1	IFNA10	ERVW-1	DHDH
109	SATL1	PPP1R3F	LDHB	UNC93A
110	YME1L1	ATP6AP1	ARPC5L	GPRIN1
111	GUF1	FUT2	CFL1	CTAG2
112	GPR119	GUCA1C	GPR141	PRR25
113	AMPD1	CC2D2A	PLXNB3	NCR3LG1
114	CYP2A7	LCE6A	ZNF208	GLT6D1
115	APLF	IDO2	L1TD1	PSORS1C2
116	XRCC4	SRA1	AKR1E2	SLC22A14
117	HLA-B	ATP8B4	SVOPL	DMD
118	HLA-G	ERVW-1	SRPK3	OR52N4
119	HLA-A	ARPC5L	MAGEC1	TMPRSS3
120	GRIA1	CFL1	PRR21	USP11
121	OBSCN	GPR141	MICA	C1orf194
122	POMT1	PLXNB3	CCDC57	GUF1
123	DMD	ZNF208	MAP3K15	SATL1
124	PRPS1L1	L1TD1	CYP2A7	ANO7
125	GLUD2	AKR1E2	UPP2	CHMP4C
126	ERVW-1	SVOPL	GRK4	KISS1
127	SLC25A43	SRPK3	CCHCR1	KRT31
128	PAICS	MAGEC1	PRIM2	KRT37

129	MICA	PRR21	ABCB5	GPRC6A
130	NCR3LG1	MICA	GPR112	GRP
131	GPR142	CCDC57	POMT1	CYP2C18
132	DNAH11	MAP3K15	ZNF681	MUC20
133	L1TD1	CYP2A7	PAICS	MUC7
134	DPPA4	UPP2	LOXHD1	P2RY4
135	MTMR8	CCHCR1	ZNF157	TAS2R46
136	ABCC12	PRIM2	ZNF880	DNAH14
137	SPERT	ABCB5	YME1L1	NOD2
138	BEST3	GPR112	GAGE10	PSCA
139	UBE2W	POMT1	OR13A1	CEACAM7
140	SPSB2	ZNF681	ANKMY1	ALDH1B1
141	ZNF645	PAICS	SLFN12L	TRAPPC2
142	ZNF157	LOXHD1	MLKL	TRAPPC2L
143	USP11	ZNF157	DNAH14	LDHB
144	ZBP1	ZNF880	OBSCN	PRPS1L1
145	KRT83	YME1L1	ZBP1	FLG2
146	KRT26	GAGE10	TRAPPC2L	FOLR3
147	KRT24	OR13A1	ATP6V1B1	CHIT1
148	KRT31	ANKMY1	FUT6	GP6
149	KRT37	SLFN12L	MMP2	FCGR2A
150	GPR141	MLKL	NPPA	PHKA2
151	IDO2	OBSCN	CEP89	LILRA1
152	INMT	ZBP1	LCE1D	MMP2
153	GJE1	ATP6V1B1	FMO2	AMPD1
154	PDE6B	FUT6	INMT	GLUD2
155	ATP8B4	NPPA	KRT37	APLF
156	FRMPD3	CEP89	CEACAM7	CHD1L
157	SVOPL	LCE1D	GRP	EME2
158	UNC93A	FMO2	CPNE1	POLN
159	EME2	INMT	MMP19	GRK4
160	POLN	CPNE1	ZNF645	TLR5
161	COL16A1	MMP19	CHIT1	MAP3K15
162	FLG2	PTH2R	PTH2R	NPPA
163	FOLR3	FRMPD3	FRMPD3	ATP1B4
164	CHIT1	ATP1B4	ATP1B4	ABCC12
165	GAGE10	PSG9	PSG9	MTMR6
166	ACSL4	ACTR3C	ACTR3C	GJB7
167	WDR63	GBGT1	GBGT1	MTMR8
168	GJB4	LCP1	LCP1	AKAP3
169	LDHB	SEMA4C	SEMA4C	GJB4
170	ASB15	DPPA4	DPPA4	GPR112
171	MAGEC1	DHDH	DHDH	CLEC7A

172	CTAG2	PSG8	PSG8	GPR141
173	IFNA10	UNC93A	UNC93A	PSG9
174	FCGR2A	FAAH	FAAH	PSG8
175	FAAH	OXSM	OXSM	FAAH
176	PPP1R3F	C4orf17	C4orf17	COL16A1
177	TLR5	GPRIN1	GPRIN1	IFNA10
178	P2RY4	CTAG2	CTAG2	LILRB4
179	TAS2R46	ABCC12	ABCC12	OBSCN
180	PNLIPRP3	PRR25	PRR25	OR7G3
181	CYP2C18	MTMR6	MTMR6	SPERT
182	KLHL34	NCR3LG1	NCR3LG1	BEST3
183	PSG8	GLT6D1	GLT6D1	ZNF208
184	ATP1B4	GJB7	GJB7	PTH2R
185	PSCA	ALDH1A2	ALDH1A2	GAGE10
186	CEACAM7	OR7G3	OR7G3	ALDH1A2
187	GJB7	PSORS1C2	PSORS1C2	GBGT1
188	CXCR3	MTMR8	MTMR8	BTNL2
189	CCDC102B	AKAP3	AKAP3	DYX1C1
190	GPR112	SLC22A14	SLC22A14	TCTEX1D1
191	FAM187B	IFNE	IFNE	CPNE1
192	PLA2G2C	GJB4	GJB4	CCDC102B
193	HLA-DQB1	PLA2G2C	PLA2G2C	SRA1
194	HLA-DRB5	DMD	DMD	GUCA1C
195	HLA-DPB1	OR52N4	OR52N4	CYP2A7
196	HLA-DQA1	DYX1C1	DYX1C1	PLA2G2C
197	TRIM48	TMPRSS3	TMPRSS3	UPP2
198	NT5C1B	USP11	USP11	C4orf17
199	CMA1	C1orf194	C1orf194	PRIM2
200	OXSM	CMA1	CMA1	PAICS
201	TRAPPC2	GUF1	GUF1	FMO2
202	TRAPPC2L	RPS6KA6	RPS6KA6	KLHL34
203	PSG1	SATL1	SATL1	IFNE
204	SRPK3	CCDC102B	CCDC102B	CMA1
205	GPRIN1	ANO7	ANO7	OXSM
206	RPS6KA6	TCTEX1D1	TCTEX1D1	MMP19
207	A2M	KLHL34	KLHL34	CELA1
208	MAP3K15	BEST3	BEST3	RPS6KA6
209	IFNE	SPERT	SPERT	ERVW-1
210	GRK4	CHMP4C	CHMP4C	ZBP1
211	MMP19	CELA1	CELA1	SLFN12L
